# Investigation of Biases and Compensatory Strategies Using a Probabilistic Variant of the Wisconsin Card Sorting Test

**DOI:** 10.3389/fpsyg.2016.00017

**Published:** 2016-01-22

**Authors:** Alexis B. Craig, Matthew E. Phillips, Andrew Zaldivar, Rajan Bhattacharyya, Jeffrey L. Krichmar

**Affiliations:** ^1^Department of Cognitive Sciences, University of California, IrvineIrvine, CA, USA; ^2^HRL Laboratories, LLCMalibu, CA, USA; ^3^Department of Computer Science, University of California, IrvineIrvine, CA, USA

**Keywords:** decision-making, Wisconsin Card Sorting Test, cognitive biases, probability matching, uncertainty

## Abstract

The Wisconsin Card Sorting Test (WCST) evaluates a subject’s ability to shift to a new pattern of behavior in response to the presentation of unexpected negative feedback. The present study introduces a novel version of the traditional WCST by integrating a probabilistic component into its traditional rule shifting to add uncertainty to the task, as well as the option to forage for information during any particular trial. These changes transformed a task that is trivial for neurotypical individuals into a challenging environment useful for evaluating biases and compensatory strategizing. Sixty subjects performed the probabilistic WCST at four uncertainty levels to determine the effect of uncertainty on subject performance and strategy. Results revealed that increasing the level of uncertainty during a run of trials correlated with a reduction in rational strategizing in favor of both random choice and information foraging, evoking biases and suboptimal strategies such as satisfaction of search, negativity bias, and probability matching.

## Introduction

Dating back to 1948, David Grant and Esta Berg’s Wisconsin Card Sorting Test (WCST) is a task that is commonly used in assessing the ability to “set-shift,” or change one’s way of thinking in the face of new goals or stimuli (Grant and Berg, 1948; Bishara et al., 2010). This task is useful in studying, modeling, and diagnosing disorders in higher-level processing areas of the brain such as the prefrontal cortex (Milner, 1963; Robinson et al., 1980; Dehaene and Changeux, 1991; Rougier and O’Reilly, 2002; Lie et al., 2006; Nyhus and Barceló, 2009). In the WCST, a subject is presented with one reference card and three to four choice cards. Each card contains an image with a particular shape, color, and number of items, and is designed such that each choice card’s feature expressions are mutually exclusive. Every choice card matches a different feature of the reference card. In each trial, one feature is selected as the “rule,” and the objective is to select a card that matches the rule for the reference card. For example, if the rule is green, the correct choice would be the card that contained green items, irrespective of the number or shape of items on that card. The WCST consists of several iterations of trials that use the same rule, followed by a rule shift that requires subjects to change their behavior.

With the goal of evoking and quantifying changes in strategizing, behavior, and biases due to uncertainty, we developed a modified version of the WCST called the probabilistic WCST (pWCST). pWCST incorporates an element of uncertainty in the form of a probabilistic rule selection, and an option to forage for information by observing a trial. Each trial has a set of three probabilities corresponding to the likelihood that a particular feature will be the rule. For example, the rule could be dictated by a 90% chance of shape, 7% chance of color, and 3% chance of number of items. These probability distributions are referred to as the Top, Middle, and Bottom rules, respectively, and these base percentages are referred to as the “ground truth probability distribution” in the paper. Because humans are oftentimes shown to make irrational decisions regarding probabilistic assessment in the face of uncertainty, incorporating a varying degree of uncertainty into the WCST adapts the task into a tool that can be used to evoke and quantify the degree of change in behavior that is introduced into the decision-making process for unpredictable events. We hypothesize that by increasing the level of uncertainty in the WCST task, we will evoke biases and strategic changes in subjects that correlate to the degree of uncertainty.

Card-based tasks in the past have been common for assessing the patterns of balancing exploration and exploitation (Hoehn et al., 2005; Worthy et al., 2007; Sang et al., 2011), both for testing diminishing resources as would be experienced in real world explore/exploit tasks, and for understanding the underlying probability in action choices. The element of information foraging is commonly studied using decision-making experiments, often utilizing a probabilistic task, although to our knowledge, the WCST has not been previously modified to accommodate this mechanism. Introducing uncertainty into the WCST has been explored in a previous study by Wilson and Niv (2012). Wilson and Niv (2012) used the WCST in conjunction with a Bayesian model in order to examine the methods by which humans decide what information to learn in a changing environment.

The present study moves beyond Wilson and Niv’s paradigm with the addition to “Observe” a trial (i.e., to collect information without affecting one’s score). While Wilson and Niv kept task uncertainty fixed, the present study investigates the effect of a variable level of uncertainty, which is hypothesized to affect both Observe behavior and strategy usage. The Observe feature introduced in the pWCST allows the subject to explore potential payoffs rather than exploit immediate gains. This adds an alternate option, similar to no-choice utility (Howes et al., 2015), to the classical explore/exploit tradeoff where subjects can practice alternate strategies in a subsequent choice phase. Another recent similar task of note is the probabilistic lights task utilized by Navarro and Newell testing the theory that humans tend to assume a higher underlying rate of change than the ground truth probability distribution during a probabilistic task (2014). In this task, subjects were told to predict which of two lights would come on, given that only one would light up on each trial. In the dynamic condition, subjects were told that the bias on these two lights could randomly change, and in the static condition, the bias was to stay the same. In both conditions, the bias was always 70 and 30%, with a 1.6% chance of switching in the dynamic condition. Subjects were allowed to either Observe or Bet on each trial, with Observe allowing them to test their response without gaining or losing points, while Betting would result in a change in points. Results showed that in both static and dynamic conditions, subjects significantly overestimated the amount of switching that occurred, which confirmed the hypothesis and suggested that it was more costly to underestimate a rate of change than overestimate. Similarly to the pWCST, subjects were allowed to Observe trials without any point gain or loss, as an alternative to betting their real points. While Navarro and Newell’s task adequately probed subject Observe behavior, the pWCST takes this task paradigm further in the inclusion of differing probability distributions, adding a level of uncertainty that cannot be directly tested in the probabilistic lights task. Additionally, the introduction of varying probability levels allows for the investigation of effects due to probability magnitudes, potentially identifying overweighting and underweighting effects, as well as differences in the level of Observe reliance.

Cognitive biases are deviations from normative strategies that occur both consciously and unconsciously in human decision making to quickly and efficiently cope with uncertainty or task difficulty. While these biases can lead to non-optimal action decisions (Tversky and Kahneman, 1974) it has also been shown that such biases, under the right circumstances, can result in near-optimal task performance, designating them, not as irrational, but as “bounded rational” behavior (Gigerenzer and Goldstein, 1996). Therefore, these biases are interesting to study both for their commentary on shortcomings of human decision-making, as well as their insight into conscious and subconscious techniques that allow for fast and frugal yet high-yielding processes for creating action decisions. The decision-making behaviors of interest, including cognitive biases, heuristics, and non-optimal/bounded rational strategies, in the present paper include negativity bias, probability matching, and satisfaction of search, all of which the pWCST is expected to evoke, that would not be expected in the traditional WCST.

Negativity bias is the unbalanced increase in salience of negative over positive feedback (Ito et al., 1998; Carretié et al., 2001; Rozin and Royzman, 2001; Vaish et al., 2008). Negativity bias may have adapted by allowing humans to focus more on information that was potentially harmful rather than helpful, as neglecting harmful information is more likely to shorten one’s lifespan. However, the presence of negativity bias, through prioritizing avoiding negative behavior patterns, could potentially delay or prevent time spent on the development of positive behavior patterns. The present pWCST is hypothesized to evoke negativity bias while WCST would not, as pWCST is inherently more challenging due to the probabilistic rule, and in higher uncertainty levels, is expected to lead to higher amounts of negative feedback. This may result in subjects spending more time foraging for information when they feel they are receiving too much negative feedback resulting from their choices.

In probability matching behavior, an individual will perform actions that roughly mirror the underlying probability structure inherent in a task environment (Vulkan, 2000; Shanks et al., 2002; Wozny et al., 2010). Probability matching can occur in situations where it is advantageous to explore options rather than exploit the best choice. When a subject feels uncomfortable with their ability to identify and exploit the most valuable option, subjects may revert to probabilistic search for information about the rules of their present task (Gaissmaier and Schooler, 2008). Probability matching is another suboptimal decision framework that pWCST is expected to evoke where WCST would not, as it is a behavioral strategy that is employed during situations in which the subject receives probabilistic payoff, a novel inclusion in the pWCST paradigm. We hypothesize that subjects will utilize probability matching as a means to cope with the uncertain environment in the pWCST, rather than the optimal strategy of continuously selecting the highest probability feature. During periods of moderate uncertainty, we expect subjects to use the Observe option to derive the “expected uncertainty” from a block of trials (Yu and Dayan, 2005).

In satisfaction of search, the individual possesses a threshold at which they determine they have collected enough information for their task (Fleck et al., 2010; Simons, 2010). As confirmatory evidence is acquired, less evidence is required from the information foraging process, which is a heuristic that can save time and energy but is not inherently rational (Gigerenzer and Goldstein, 1996). Having preconceived notions about the underlying nature of a task is in itself a bias, and when those notions are accompanied by confirming evidence that is determined by a probability rather than a static metric, these biases become compounded. We hypothesize that, during higher uncertainty levels, the satisfaction of search threshold will be higher than in lower uncertainty levels. This may be due to a resulting increase of conflicting information necessitating a larger sample size to reach the same level of confidence. The pWCST allows for the opportunity to study satisfaction of search where the WCST would not, owing to the inclusion of an option that allows for risk-free information foraging. Through the incorporation of the Observe option, we predict that the satisfaction of search threshold (i.e., the number of Observe trials necessary for a subject to be confident enough in their rule beliefs to cease foraging) will be higher under higher uncertainty conditions.

Win-Stay-Lose-Shift (WSLS), a strategy commonly used in game theory (Nowak and Sigmund, 1993; Imhof et al., 2007) involving staying with an action following its successful use and shifting to another action following its unsuccessful use, was selected for strategic assessment alongside probability matching during the pWCST. These strategies were chosen for comparative analysis in order to provide reasonable baselines for subject performance that realistically encompassed biases, suboptimal strategizing, and human limitations. While probability matching and WSLS behaviors are formal strategies that lead to positive performance in the pWCST, the pWCST is akin to a multi-armed bandit task (Lee et al., 2011) coupled with the WCST as a result of the introduced probabilistic component. In the pWCST, optimal task performance consists of always selecting the highest probability feature.

## Materials and Methods

### Human Participants

Sixty subjects (ages 18–25) were recruited in two sessions of 30 subjects through an online database maintained by the Experimental Social Science Laboratory (ESSL) at the University of California, Irvine (UCI). This database is comprised of UCI’s undergraduate and graduate student population that have agreed to participate in computer experiments based in social and economic decision-making conducted by members of the School of Social Sciences and affiliated organizations. Subjects were not selected using background characteristics (age, race, gender) other than their student status. Subjects participating in the second session were prescreened to ensure that they had not previously participated in the experiment. Experimental protocol was reviewed and approved by the UCI Institutional Review Board, and informed consent was obtained from all participants.

### Experimental Design

Subject data was collected using desktop PCs within the ESSL. Prior to the experiment, subjects were instructed on the basic structure of the task, the payment system consisting of a baseline amount plus an incentive sum reliant on their performance, and their right to cease participation without penalty for any reason at any time. Subjects then participated in two behavioral tasks, the pWCST and a version of the Wason Selection Task, a similar decision-making task also investigating biases and compensatory strategizing related to uncertainty, in a randomly assigned order. The Wason task will not be discussed further in this paper, as it is the subject of a separate analysis. Each behavioral task incorporated a brief tutorial to train subjects on the tasks, as well as inform them that the rule involved in the tasks would change throughout the experiment. Subjects were not, however, informed that the rule sets they would encounter would be probabilistic, in an effort to preserve unprejudiced strategizing in the face of unreliable feedback.

Upon completion of both tasks, subjects received a $7 flat rate for participation, as well as compensation dependent on their performance in both tasks. The rate of compensation for WCST was $0.02 per point, with a minimum performance-based payout of $2. Total payments ranged from $10 to $30.

### Probabilistic Wisconsin Card Sorting Test (pWCST)

Subjects played a total of 550 pWCST games, which were split into 11 blocks of 50 games each. There were four block types – No, Low, Moderate, and High uncertainty – all of which were presented three times each except High, which was only presented twice as a result of the block order. The probability sets associated with each block type are listed in **Table 1**, and the order of the blocks is listed in **Table 2**. The first third of the condition presentation order was designed to scale up the uncertainty-based difficulty gradually in order to investigate the effect of increasing uncertainty on strategy choice, information foraging, and the appearance of biases. As stated in the hypotheses, it was expected that altering the underlying feature probabilities and increasing uncertainty would lead to an increase in information foraging and satisfaction of search threshold, as well as the emergence of negativity bias. The second third of the order was intended to assess the rate at which subjects would adapt to a gradually decreasing uncertainty under their previous expectation derived from the trend of increasing uncertainty, which was expected to reverse the hypothesized increase in information foraging behavior and other changes in formal strategizing. The final third of the condition order was used to assist in testing for order effects and to incorporate more unpredictable jumps in uncertainty level between blocks.

**Table 1:** Probability sets for Wisconsin Card Sorting Test (WCST).

Uncertainty	Top rule	Middle rule	Bottom rule
No	100%	0%	0%
Low	90%	7%	3%
Moderate	75%	20%	5%
High	60%	30%	10%

**Table 2:** Block order/Criterion for WCST.

Uncertainty	Block order	Criterion
No	1, 7, 10	90% correct
Low	2, 6, 11	80% correct
Moderate	3, 5, 8	65% correct
High	4, 9	50% correct

**FIGURE 1:**
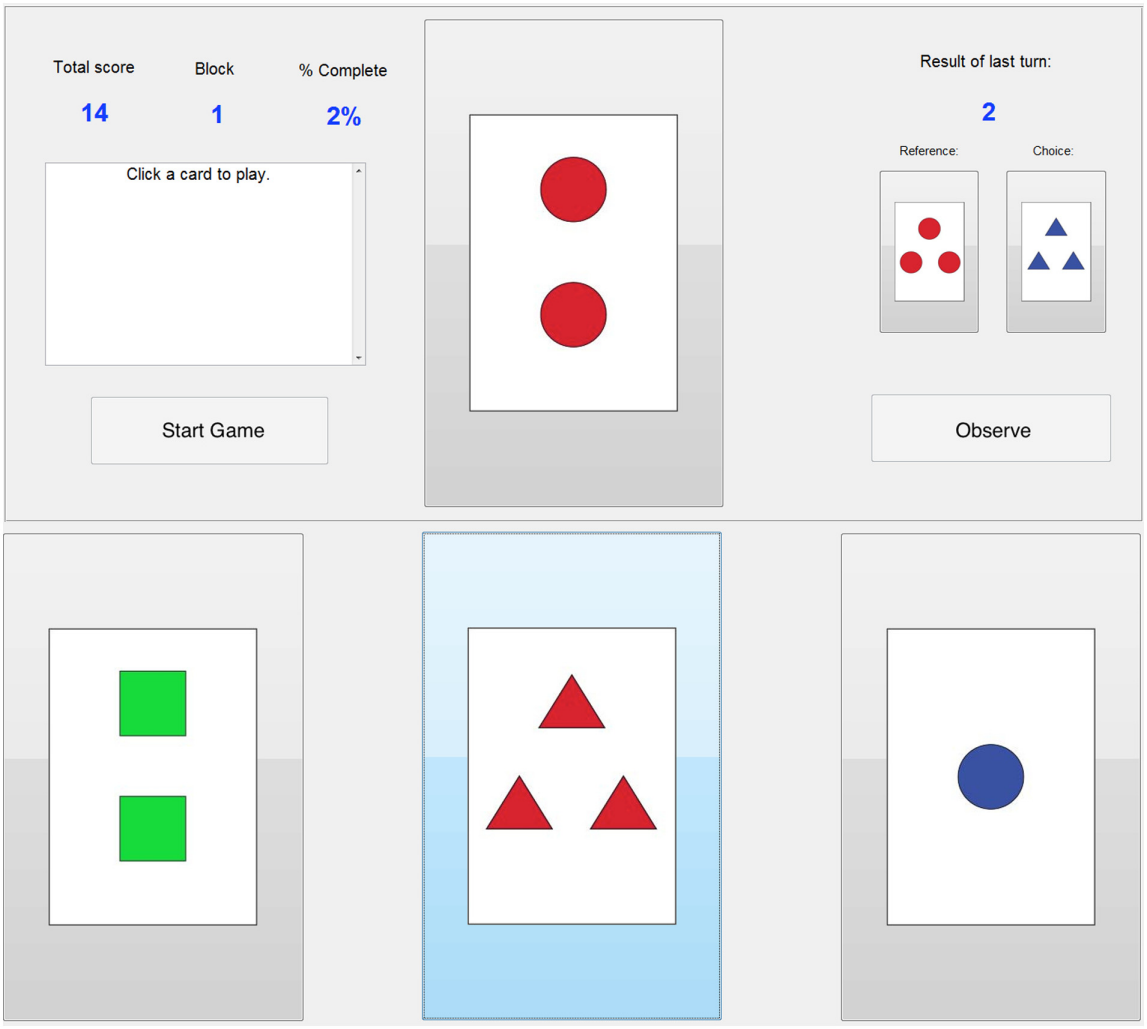
**Screenshot of probabilistic WCST (pWCST) GUI**. On a given trial, subjects would choose between one of the three cards on the bottom, attempting to match the rule of common feature between the reference card and the target. Two points were received for each choice matching the rule, and two points were taken for any choice that did not match the rule on that given trial. Alternatively, subjects could click the Observe button in order to simulate a trial, in which a target card was randomly chosen and presented to the subject along with the score they would have received had they chosen it, with no actual point gain or loss.

Subjects were required to choose a feature (color, numerosity, or shape) of the presented cards that they believed to be in compliance with a particular rule (**Figure 1**). Each feature in a rule set had a probability associated with it. A feature set was randomly assigned to a probability at the beginning of the experiment and again each time a criterion for successful trials was met. This criterion was a percentage of correct answers out of the most recent 10 trials (see **Table 2**). For example, in a Low uncertainty block, subjects who chose correctly on at least 8 of 10 trials received a rule change. To reduce the predictability of set shifts, assessments did not begin until a randomly selected number of trials (i.e., between 10 and 15) had elapsed since the last block change or feature set shift.

In an effort to assess subjects’ preference for foraging behavior to cope with the uncertainty of their task, subjects had the option to “Observe” rather than choosing one of the three cards during each trial. In this case, the subject forewent selecting one of the choice cards for that trial by observing, at which point a choice card was selected at random and the subject was informed what the card was and whether or not it followed the rule. By using Observe, subjects had a chance of collecting information about the rule until they were able to reduce their own level of uncertainty enough to select cards on their own. In essence, an Observe trial could be used to obtain information, but resulted in a loss of potential points. Alternatively, an Observe trial could be used to spare the subject from losses. The first block did not feature the Observe option, as it was meant to provide subjects with practice for the fundamental task and to determine whether they had fully understood the instructions.

The pWCST software interface (**Figure 1**) consisted of a reference card and three clickable cards that subjects chose from during each game. If a subject selected a card that matched the correct feature of the reference card, they received two points. Two points were taken away for each incorrect answer. The graphical user interface allowed subjects to see the percent of the task that they had completed, the block they were in, their total score, the reference and chosen card from the previous game, and the points received on the previous game. Subjects advanced through trials at their own pace, with the majority finishing both behavioral tasks in 60–90 min.

Unless otherwise specified, all reported *p*-values were derived using the two-sample Kolmogorov–Smirnov hypothesis test (refer to MATLAB kstest2). Because these *p*-values were based on multiple comparisons, the significance threshold was Bonferroni corrected by dividing 0.05 by the number of comparisons.

## Results

### Score

**FIGURE 2:**
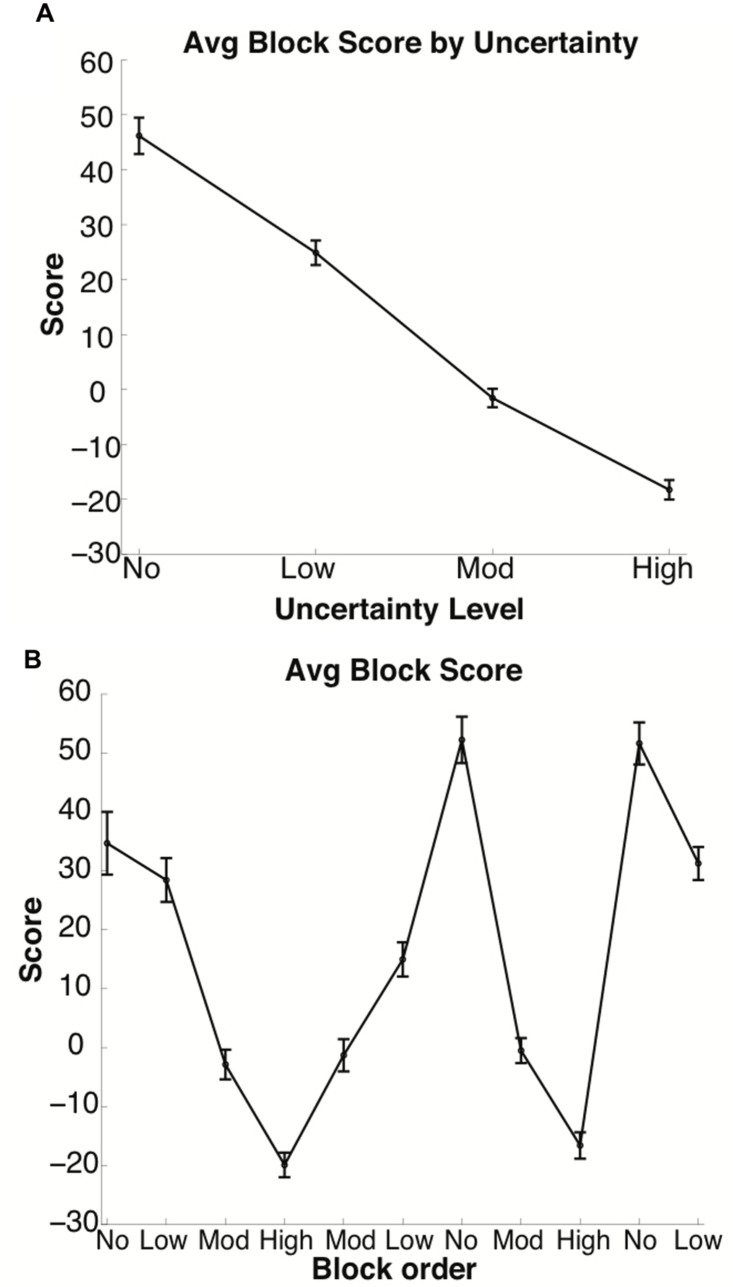
**Average score per block by uncertainty**. Average score decreased as the block uncertainty level increased. In **(A)**, the *x*-axis is grouped by uncertainty level. In **(B)**, blocks along the *x*-axis are arranged in the order in which they were presented to subjects. Bars denote the standard error.

Subject performance varied depending on the level of uncertainty, as revealed by the average score per uncertainty level [see **Figure 2A** and Supplementary Tables S1–S3] and average score per block (see **Figure 2B** and Supplementary Tables S4–S6]. The score per block averaged over all 60 subjects was significantly different between the four uncertainty levels (*p* < 0.001), with higher uncertainty associated with lower, often negative, scores.

### Observe Use

**FIGURE 3:**
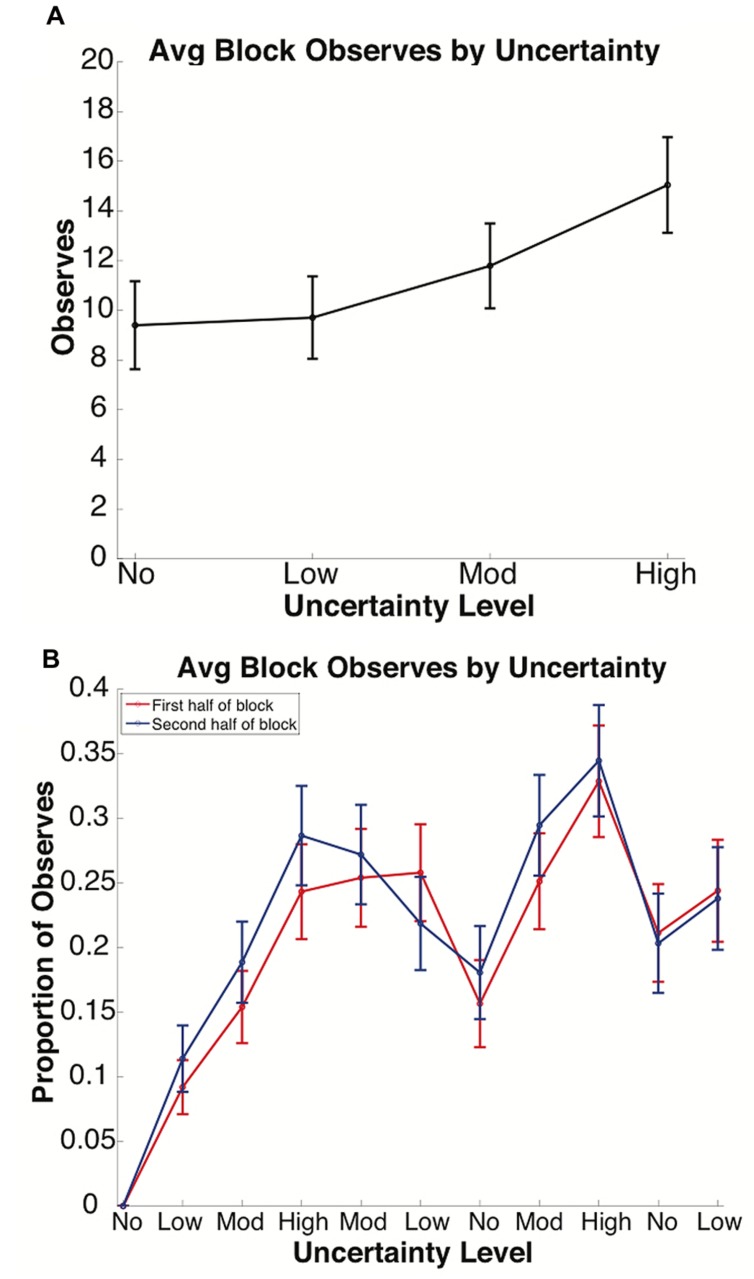
**Average Observe use per block by uncertainty. (A)** Average Observe usage increased alongside increasing block uncertainty. **(B)** Observe usage per half-block by uncertainty. Observe usage largely increased during the second half of each block. This gap increased along with increasing uncertainty and decreased with decreasing uncertainty, in some blocks even causing usage in the first half to overtake the second half. In **(A)**, the *x*-axis is grouped by uncertainty level. In **(B)**, blocks along the *x*-axis are arranged in the order in which they were presented to subjects. Bars denote the standard error.

We analyzed Observe use in order to quantify how changes in uncertainty level could affect satisfaction of search thresholds. Search behavior, or information foraging, was assessed as use of the Observe option, a means of collecting information regarding the feature rule without risking points. Typical subject behavior was altered by changes in uncertainty level, as revealed by the number of times the Observe option was used in blocks of each uncertainty level (see **Figure 3** and Supplementary Tables S7–S9). The number of trials in which Observe was used in a block increased with uncertainty level (see **Figure 3A**), and comparisons of No vs. Moderate (*p* = 0.006), No vs. High (*p* < 0.001) and Low vs. High (*p* = 0.002) uncertainty were found to be significant. To examine how subjects used Observe to gain knowledge about the task structure, we compared the amount of Observe trials during the first and second halves (25 trials each) of each block (see **Figure 3B** and Supplementary Table S10). Although no comparisons between the first and second halves of a block were shown to be significant, there was an existing trend indicating that Observe trials were more common in the second half than the first half of a block in most blocks, suggesting a subject preference to perform their own sampling within a new block before resorting to Observe use. This difference in Observe usage increased alongside increasing uncertainty and decreased with decreasing uncertainty. Taking the number of Observes per block as insight into information foraging behavior, these results support the hypothesized increase in the satisfaction of search threshold at higher uncertainty levels.

**FIGURE 4:**
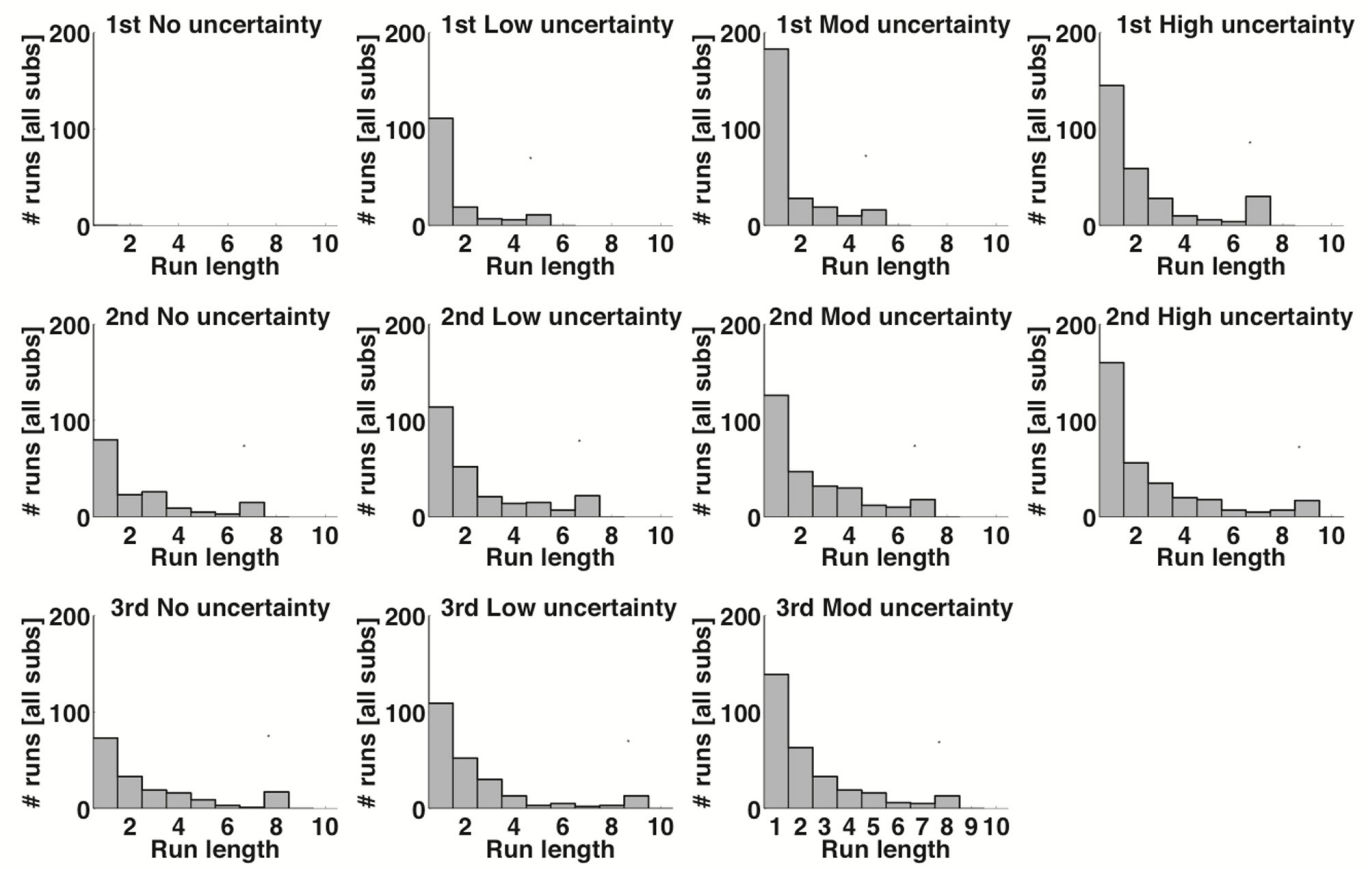
**Run length of Observe usage by all subjects per block**. Blocks are grouped horizontally by presentation number of each uncertainty level, and vertically by uncertainty level. The title numbers indicate the true block order as experienced by the subjects. The length of the tail, and thus the average length of runs of Observe trials, increased both with increasing uncertainty and successive presentations of each uncertainty level.

To further measure how Observe usage changed over time and uncertainty level, we measured the runs of consecutive Observe usage (see **Figure 4** and Supplementary Tables S11–S16), which here is defined by the number of trials in a row a subject chose to Observe rather than picking one of the three cards. As shown in **Figure 4**, average number of Observe runs increased with both uncertainty (compare rows of **Figure 4**) and time (compare columns of **Figure 4**), although the only comparison that reached significance under a two-sample Kolmogorov–Smirnov test was that of the first and second presentations of the Low condition (Blocks 2 and 6 in **Figure 4**; *p* < 0.015). In accordance with the previously discussed results, this increase in runs of Observe usage also comments on satisfaction of search, which rose over time alongside score (see **Figure 2B**), suggesting more accurate performance during non-Observe trials based on a more extensive collection of information over the length of a block.

### Win-Stay-Lose-Shift (WSLS) Strategy

**FIGURE 5:**
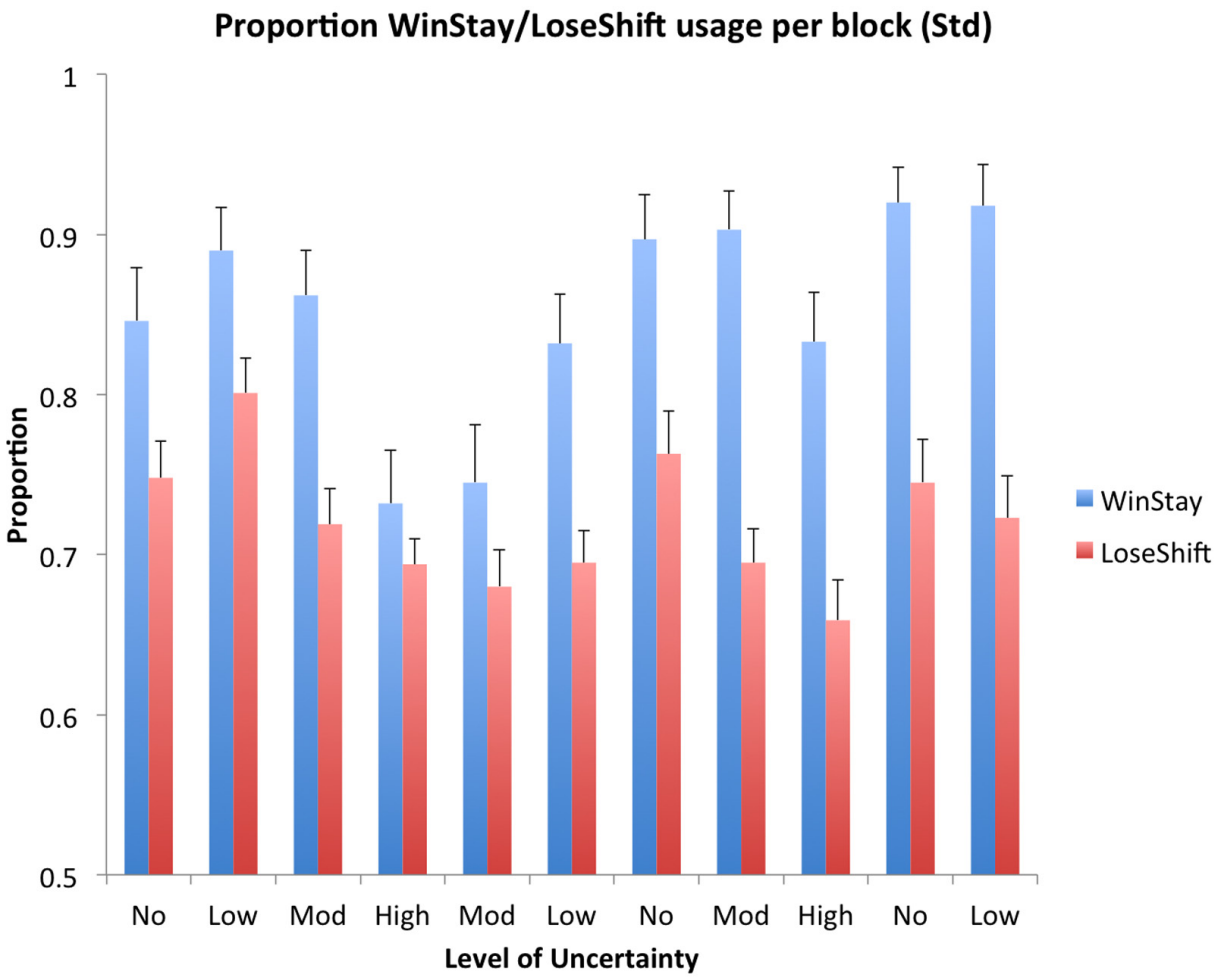
**Win-Stay and Lose-Shift use by uncertainty**. “Win-Stay” bars denote the percentage of Win-Stay behavior out of all trials in which the subject chose the correct rule, while “Lose-Shift” bars denote the percentage of Lose-Shift behavior out of all trials in which the subject chose incorrectly.Bars denote the standard error and *x*-axis is arranged in the order the blocks were presented (**Table 2**).

Win-Stay-Lose-Shift usage was sensitive to the level of uncertainty and the order of blocks. The uncertainty level and the block order affected the use of Win-Stay and Lose-Shift behavior (**Figure 5** and Supplementary Tables S17–S28). The significant dominant strategy was found to be Win-Stay (i.e., choosing the same feature after a win), for win trials and Lose-Shift (i.e., choosing a different feature after a loss) for lose trials over all blocks (*p* < 0.001). While no significant order effects exist, a trend of increasing Win-Stay usage over time for all uncertainty levels was observed, excepting for the second Moderate and Low blocks. On average, Win-Stay and Lose-Shift strategy use decreased with increasing uncertainty, and this effect was significant for comparisons between No and Moderate, No and High, and Low and High uncertainty levels for both Win-Stay and Lose-Shift, as well as Low and Moderate Win-Stay (Lose-Shift Low vs. High: *p* < 0.006; all others: *p* < 0.001).

For some comparisons, blocks of high uncertainty were found to bias future behavior. The proportion of trials in which a Win-Stay strategy was used in the second Moderate and Low uncertainty blocks was lower than that of the other presentations of Moderate and Low uncertainty blocks (see **Figure 5**). This effect was found to be significant in the comparison between the second and third presentations of the Moderate uncertainty level (*p* < 0.001), and was expected as a likely consequence of the experienced high uncertainty in the High block persisting to devalue reliability of confirmatory evidence in proceeding blocks, coupled with the subjects’ lack of knowledge that the uncertainty level would decrease rather that increase over time.

A similar order effect was found in Lose-Shift strategy. However, in contrast to Win-Stay, the proportion of Lose-Shift trials did not show as dramatic of a change over time between presentations of the same uncertainty level (**Figure 5**). There was a significant decrease in Lose-Shift usage from the first to second presentations in Low uncertainty blocks (*p* < 0.001). There was also a substantial, but not statistically significant, decrease in Lose-Shift usage from the first to the second presentations of the Moderate uncertainty blocks. This behavior, similar to patterns of usage for Win-Stay, appears to be a consequence of lowered informational reliability from the High uncertainty block.

### Probability Matching

**FIGURE 6:**
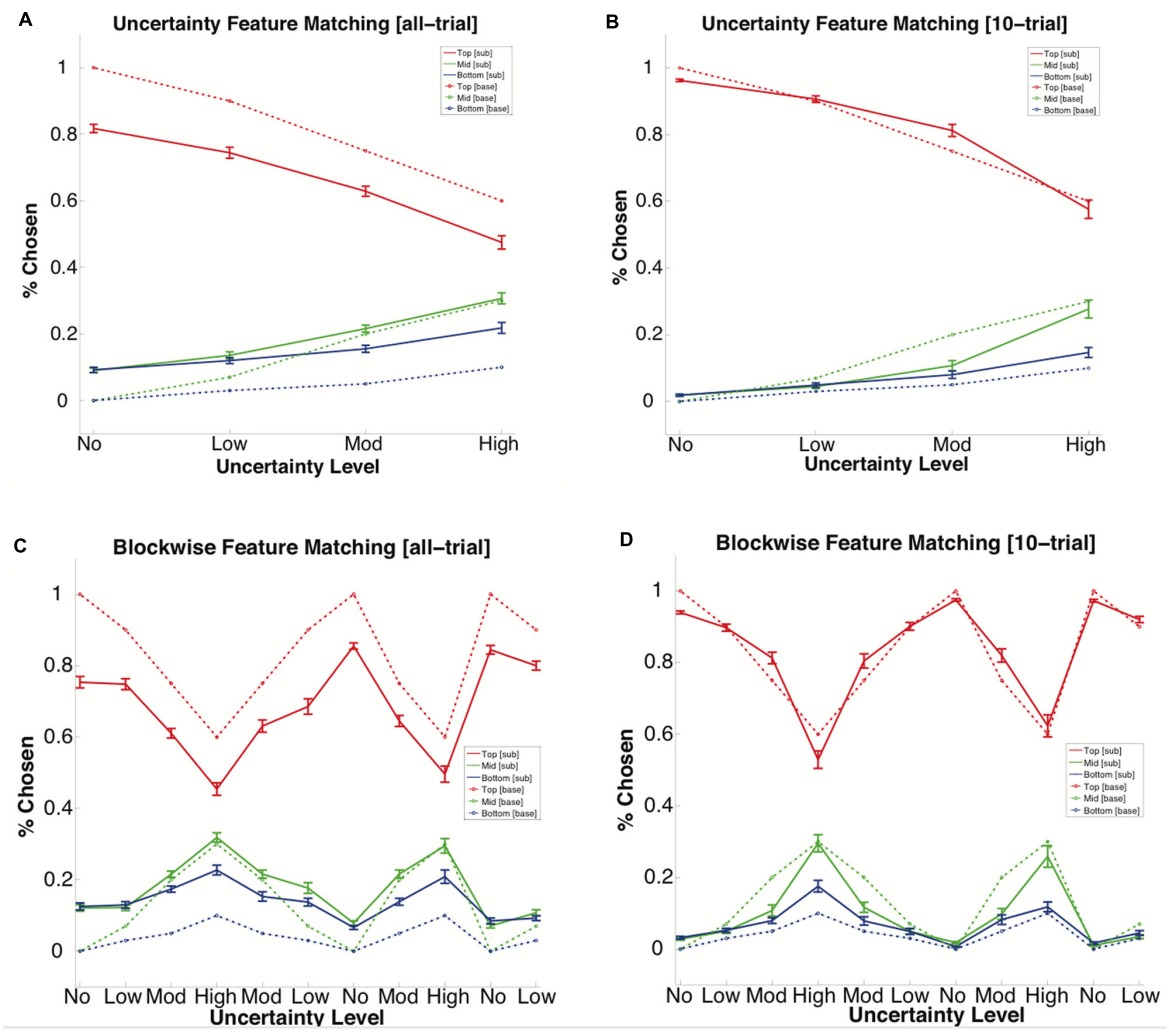
**Subject feature matching vs. ground truth probability distribution averaged over all non-Observe trials for all blocks of each uncertainty level for all subjects**. Solid lines indicate subjects’ choice percentages for the Top, Middle, and Bottom probability rules averaged over all trials of the No, Low, Mod, and High conditions. The dashed lines indicate the base rule frequency of the Top, Middle, and Bottom rules during each condition (see **Table 1**). **(A)** Feature matching percentages averaged over all non-Observe trials for each uncertainty level. **(B)** Feature matching percentages averaged over the last 10 trials (excluding Observes) before a rule shift (i.e., probability set to feature matchup scrambled) for each uncertainty level. **(C)** Feature matching percentages averaged over all non-Observe trials for each block. **(D)** Feature matching percentages averaged over the last 10 trials (excluding Observes) before a rule shift. Bars denote the standard error.

We analyzed subjects’ choice behavior to see if they attempted to match the underlying distribution of rules rather than a normative strategy such as always choosing the most likely feature. Subject feature selection was averaged over each block to derive rule choice percentages. Feature selection percentages were calculated by dividing the number of times subjects selected the Top, Middle, and Bottom probability features by the number of non-Observe trials per block to obtain proportions (**Figures 6A,C**, and Supplementary Tables S29 and S30). An additional analysis was conducted that used only the last 10 trials excluding Observes before a rule shift using the same metric, the window imposed by the rule shifting mechanism’s threshold for analyzing correct responses (**Figures 6B,D**, and Supplementary Tables S31 and S32). These data were compared with the ground truth probability distributions that were established prior to the experiment (see **Table 1**). Results showed that the probability of selecting the Top, Middle, and Bottom feature roughly followed the ground truth probability distribution. Rather than choosing the highest percentage feature, Top rule selection decreased with increasing uncertainty.

To further examine the use of probability matching behavior, comparisons between uncertainty levels were made on the last 10 trials. Using a one-sample *t*-test, 10-trial uncertainty-wise comparisons showed that the Top rule was significantly different from the ground truth probability distribution only during No uncertainty (*p* < 0.001), the Middle rule was significantly different from the ground truth probability distribution during No, Moderate and the third presentation of Low uncertainty (*p* < 0.001), and Bottom was significantly different from the ground truth probability distribution only during the first presentations of No, Low, and High uncertainty, as well as the third presentation of No uncertainty (*p* < 0.001; **Figure 6D**). In the 10-trial analysis, subject rule selection was only universally different from the ground truth probability distribution for all rules during the No uncertainty condition. Taken together, these results suggest that subjects did tend to probability match rather than an optimal Top rule selection.

### Over/Under-Selection

Overselection, in the case of this experiment, is defined as choosing low probability rules at a significantly higher probability than the ground truth, and underselection is similarly defined as choosing high probability rules to a less often than the ground truth. We analyzed all trials and 10-trial block-wise data for similarity to ground truth. Using a one-sample *t*-test, all-trial block-wise rule selection proportions, with the exception of Middle/High, were significantly different from base rule percentages (Mid/Mod1: *p* < 0.005, Mid/Mod2: *p* < 0.005, Mid/Mod3 < 0.005; all others: *p* < 0.001; **Figure 6C**). Subject usage of the Top rule universally fell under the ground truth, while selection of the Bottom rule was always more frequent than the ground truth. Subject usage of the Middle rule fell over ground truth, but this difference declined with increasing uncertainty until the difference was not significant. These results provide evidence for underselection of the Top probability rule alongside overselection of the Bottom probability rule.

## Discussion

In the present study, we showed that a probabilistic version of WCST with a means of information foraging is an effective tool for evaluating compensatory biases and suboptimal strategizing related to rational choice in economic decision-making. Through analysis of feature selection, it was clear that Win-Stay Lose-Shift and Probability Matching behaviors were the most prevalent strategies. The principal findings were that: (1) the threshold for *satisfaction of search* increased with uncertainty (**Figures 3** and **4**), (2) *negativity bias* occurred in trials following periods of high uncertainty (**Figures 3** and **5**), and (3) while subjects followed the trends of *probability matching* behavior, subjects also persisted in underselecting the Top probability rule while overselecting the Bottom probability rule (**Figure 6**). These findings address the relationship between uncertainty and the prevalence of biased and suboptimal selection behavior, a field with potential applications in the development of cognitive technologies and improving the decision-making techniques of human operators.

Through the incorporation of information foraging using an Observe option, satisfaction of search bias was demonstrated in the present study. The use of the Observe button increased with increasing uncertainty, indicating that the threshold for a subject’s satisfaction of search (Fleck et al., 2010), or the amount of information a subject must collect before they are confident enough to take action in their current circumstances, similarly increased as hypothesized. This increase in Observe usage can be explained both as an avoidance tactic for losing points when the rule has a high degree of ambiguity, and as an attempt to learn a probability rule by collecting more information under conditions of high uncertainty. The relatively low Observe usage in lower uncertainty blocks may be the result of a lower satisfaction of search threshold or high confidence level. When analyzing the length of trials in which Observe was used consecutively, referred to as a “run,” number of runs increased for both uncertainty level and block order (**Figure 4**). This trend also supports the increase of a subject’s satisfaction of search threshold with higher uncertainty levels, but also indicates that subjects may be attempting to prevent further losses later on in the game by Observing more. By increasing the number of Observe runs in later blocks, subjects appear to behave according to an exploration paradigm in order to ensure they have the correct rule at multiple time points before risking their point reserve by participating in trials.

In the analysis of the prevalence of Observe usage during the first and second halves of a block, it was revealed that Observe usage was typically higher during the second half of a block, although this trend reverses in trials that follow presentations of High uncertainty blocks (**Figure 3B**). It is likely that the normal trend speaks to the subjects’ preference to attempt to *exploit* their own theories in a new block before resorting to Observe usage to *explore* other options. The exception that occurred within the second presentations of Moderate and Low uncertainty blocks was likely a holdover following the first presentation of the High uncertainty block. Subjects did not yet know that uncertainty could decrease over time, and may have kept foraging for information in preparation for a higher uncertainty future. This behavior is indicative of an increase in risk aversion that led to an artificially increased level of satisfaction of search in lower uncertainty blocks as a result of the previously experienced higher uncertainty blocks. Furthermore, this result corroborates the observation that humans tend to shift to an information-seeking strategy when considering longer horizons for overall rewards (Wilson et al., 2014).

Negativity bias, or the tendency to remember negative feedback more strongly than positive (Rozin and Royzman, 2001), can be seen through increased risk-avoidance behavior that appeared after an increase in negative feedback. For example, the relatively slow (two block) return to a previous levels of WSLS following the first difficult, High uncertainty block suggests a negativity bias (see **Figure 5**) in that subjects’ decisions were less likely to be influenced by confirmatory evidence and consistently select reinforced feature choices. Furthermore, this bias led to a substantial increase in Observe usage between the first and second presentations of Moderate uncertainty and a substantial decrease in score between the first and second presentations of Low uncertainty (see **Figure 3B**). As discussed in regard to satisfaction of search, the increase in Observe usage for higher uncertainty conditions supports the hypothesized evocation of negativity bias, as subjects may have opted for observing trials after experiencing a large degree of negative feedback as a result of their choices (see **Figure 3A**). Additionally, the finding that Observe usage is higher in the second half of a block other than in the two blocks directly following the first presentation of the High uncertainty condition is in support of a negativity bias (see **Figure 3B**). After experiencing a large degree of loss during the first half of a block, a subject may have made use of the Observe option more often in the second half of the block to prevent more loss. It might be that the feeling of loss persists from the end of the High uncertainty block through the beginning of the following blocks, creating a desire to prevent further losses until the underlying probabilities of the new block are better understood. Although this trend was observed, it would be of interest to conduct a follow-up study that utilizes more blocks in order to see the shift from high to low uncertainty enough times to confirm this theory with a higher significance level.

A trend indicative of over- and under-selection emerged regarding subject choice percentages for the Top, Middle, and Bottom probability features (**Table 1**) as a factor of uncertainty level. Subjects tended to overselect the Bottom feature, and underselect the Top feature across all uncertainty levels at a relatively consistent rate (see **Figure 6**). Subjects likely experienced trials in which the Bottom feature appeared commonly enough in the small sampling of trials to lead subjects to perceive their frequency as higher than the base truth. While the explanation for this result is not unequivocally clear, there are a few potential factors that may, by themselves or collectively, have resulted in the trends seen in the data. It is possible that an effect such as representativeness, the tendency to underselect and overselect as a result of small trial size and uncertainty (Tversky and Kahneman, 1974), could have led to this result. The most conservative explanation is that this effect was caused by noise in the data (Erev et al., 1994; Hilbert, 2012; Costello and Watts, 2014), perhaps due to feature choice error or the random sampling of features in lieu of a more concrete strategy. However, due to the consistency of this effect between subjects, it is unlikely that the identified effect could be completely described by noise. Moreover, if the effect was primarily due to noise, the deviation from the ground truth probability at high uncertainty levels should have led to increased random searching, which was not the case. Building slightly from this conservative explanation, it is possible that the uncertainty itself is the reason for the trend, given that a higher level of uncertainty leads to a longer feature sampling period as it becomes more difficult to identify the Top rule while experiencing a high degree of information obfuscating belief formation. As an intermediary view between noise and bias, Gigerenzer (1991) posits the idea that, while the apparent trends in the data exist, they do not necessarily signify violations of probabilistic reasoning. In a similar view posed by Haselton, such trends are characterized as the result of “design features” rather than “design flaws” (Haselton et al., 2005). Under this notion, the tactics exhibited by the subjects instead identify fundamental properties of probability and statistical theory in a situation involving varying degrees of uncertainty in task feedback. Building upon the ideas of Simon (1972), Gigerenzer provides additional applicable work concerning the idea of “bounded rational” strategizing, or the use of strategies that can be considered rational within the confines of a task. Given the uncertainty inherent in the pWCST, it is a further possibility that the identified trend falls under the category of a bounded rational strategy (Gigerenzer and Goldstein, 1996). As discussed below, one of our future plans is to develop an optimal model that could be compared to subject behavior in the pWCST. Such a model could provide more concrete support for one of these theories in explaining under- and over-selection of rules under uncertainty.

Perhaps the most interesting aspect of the subject choice percentage data is the trend of the Middle probability feature. Initially, the Middle probability feature, much like the Bottom probability feature, is overselected. However, as the uncertainty level increases, this overselection gradually tapers off until the selection percentage nearly matches the ground truth. It is possible that this result speaks to a threshold at which a probability becomes just substantial enough that it no longer falls prey to the bias that causes it. We predict that, in the pWCST paradigm, that threshold would fall at 33%, the uniform distribution given three feature choices. In the current paradigm, the Middle feature fell just short of that in the High uncertainty condition at 30%, and as would be expected, averaged subject selection of this feature was just above the ground truth probability distribution in this uncertainty level. In order to reveal more concrete evidence for this theory, in future work, we would like to add another uncertainty level to this paradigm in which all three features are at uniform probability. It would also be helpful in the future to design a model capable of playing the pWCST in an effort to make a firm assessment of baseline performance within each probability level, given the ambiguous nature of this particular result.

Lastly, probability matching behavior (Wozny et al., 2010) was observed in the analysis of overall strategy usage. Subjects feature choices roughly mirrored the ground truth probability distribution in trend in the 10-trial analysis (**Figure 6B**), with a shift for over/underselection in the all-trials analysis (**Figure 6A**). This finding indicates that rather than utilizing the optimal strategy of always selecting the Top probability feature, subjects favored selecting the features based on what they knew of their underlying ground truth probability distribution. An alternative explanation is that the variability seen in the data is caused by feature exploration, as subjects will need to test hypotheses by switching between the features before deciding upon their most rewarding rule. In order to address this possibility, we analyzed the probability matching data for just the last 10 trials before a rule shift occurred, with the rationale being that the subject needed to reach a high percentage of correct feature choices in order to engage the rule change, suggesting that they had at that point solidified their strategy. Even in the 10-trial analysis, not only did subjects still eschew the optimal strategy in favor of probability matching, but their choices more closely matched the probability matching strategy than with the overall data. This finding provides strong evidence for the presence of this cognitive bias under varying levels of uncertainty.

There is the possibility that some of the biases found in this paper, especially the over/underselection of rules, may more conservatively fall under the explanation of noise in the data (Erev et al., 1994; Hilbert, 2012; Costello and Watts, 2014). While the explanations presented above serve as potential causes for the trends observed, we do recognize the possible effects of noise or other potential explanations for the consistent trends in behavior across subjects leading to potentially suboptimal decision-making. While we cannot know the exact strategy, motivation, and perceptual acuity the subjects exhibited during these blocks, the trends of irrational and occasionally detrimental gameplay strategies that are evident in the collected data suggest the explanation that to some degree, subjects were under the influence of biases and suboptimal strategizing that prevented them from behaving rationally as dictated by the tenets of Game Theory (Zagare, 1984). However, the not yet unambiguated nature of these conclusions invites further investigation into the underlying causes for the patterns of behavior exhibited by subjects performing the pWCST.

The present study introduces a variation of the well-known WCST to examine biases, strategy usage, and decision-making under uncertain conditions. This study sought to expand upon previous experiments using the WCST and similar decision-making tasks to investigate uncertainty-related changes in behavior in subjects. The two primary extensions to the WCST are the introduction of uncertainty in the form of probabilistic feature selection, and the option to “Observe” a trial by allowing the computer to select a card for the user, showing them the outcome with no change to their score. These initial findings suggest that the present pWCST can evoke interesting deviations from normative behaviors.

The results of the pWCST task support the assertion that under increasing degrees of uncertainty, people tend to respond with a decreasing capacity for optimal decision-making behavior. However, there are a few modifications to the present paradigm going forward that would add to its statistical power and investigative scope. In future experiments using this task, it would be desirable to query further self-report data in order to elucidate the subject’s mental state when performing the pWCST to form stronger conclusions regarding the reasons behind the biases that were evident in the data. The lack of self-report data is a limitation of the current study, and stronger conclusions might have been formed regarding biases had this data been collected. As mentioned above, we were limited by the number of uncertainty levels and recommend that future studies investigate additional uncertainty levels that staircase down to a uniform probability distribution, 33/33/33, in order to test the hypothesis that over/underestimation has a threshold, perhaps adding a shallower gradient between uncertainty levels for more accurate identification.

In regard to the discussion of over and underselection of features, the strategies taken by our subjects might be better understood if compared with an optimal computer model that played the pWCST. This is something we plan to explore in the future. Such a model would provide a better baseline for human subject performance in an effort to better assess the validity of claims about subject strategizing in the present study and might support conclusions about the presence of cognitive biases. Using performance data from the model, we would be able to comment on how each bias behaves in isolation, in the presence of other trends and biases, and the effects it would have on memory for future decision-making. Additionally, we would be able to distinguish, depending on whether the model outperformed subjects or roughly approximated their results, whether human performance on the pWCST could be considered bounded rational (Gigerenzer and Goldstein, 1996) or suboptimal. Another further analysis that could provide insight into the validity of cognitive biases in the data is minimum description length modeling (MDL; Rissanen, 1983). Typically used in order to provide support for one theory among many that exist to encapsulate a trend found in data, MDL would be well-suited for disambiguating the aforementioned results, particularly regarding the over and underselection of features, which has been shown to have varying potential explanations.

An additional bias that might fit within the scope of the paradigm is confirmation bias. Confirmation bias is the practice of seeking out information that confirms one’s prior beliefs rather than testing disproving information in an attempt to elucidate the ground truth in a situation (Nickerson, 1998; Doll et al., 2011). While the confirmation bias may have been an adaptive shortcut that increased human survival by enabling expedient development of heuristics, this bias can also lead to either incomplete or incorrect perceptions of the world in many circumstances (Klayman, 1995). We have previously shown that satisfaction of search can work in conjunction with the confirmation bias to lower the threshold at which a subject stops foraging for information (Phillips et al., 2014). This bias could be tested within pWCST by the addition of the ability to choose which feature is being used in Observe trials like in the paradigm utilized in Navarro and Newell (2014), instead of that feature being randomly selected. Confirmation bias would be a natural extension for this paradigm because, unlike the WCST, the pWCST, especially at high levels of uncertainty, would lead subjects to believe that low probability features are more common than they are as a result of conclusions based on a small sampling of trials.

Going forward, pWCST serves as a suitable platform for continuing to investigate biases and suboptimal strategies that are not commonly evoked by the traditional WCST, such as those described in this paper, as well as confirmation bias given the alterations described above. pWCST also holds potential use in investigating the tradeoff between balancing of information foraging and trial and error strategizing. The pWCST in its current form allowed for understanding when subjects were foraging for information or testing their hypotheses of the task structure. The revised Observe mechanism proposed above would make the held beliefs of the subject clearer, allowing a tighter investigation of when and how information foraging transpires. Gaining a clear understanding of the ways in which humans engage in suboptimal strategizing and the mechanisms that cause them to arise holds importance in a variety of applied positions, such as reducing human operator error, improving adaptive educational software, and modeling cognitive processes for medical and research applications.

## Conflict of Interest Statement

The authors declare that the research was conducted in the absence of any commercial or financial relationships that could be construed as a potential conflict of interest.
