# Supplementary material for: Investigation of Biases and Compensatory Strategies Using a Probabilistic Variant of the Wisconsin Card Sorting Test
Source: Front Psychol. 2016 Jan 22;7:17. doi: 10.3389/fpsyg.2016.00017 (PMC4722127; doi:10.3389/fpsyg.2016.00017)
Supplement: Supplementary file 1 [file Data_Sheet_1.DOCX]

Supplementary Material

Investigation of biases and compensatory strategies using a probabilistic variant of the Wisconsin Card Sorting Test

Alexis B. Craig*, Matthew E. Phillips, Andrew Zaldivar, Rajan Bhattacharyya, Jeffrey L. Krichmar

*** Correspondence:** Corresponding Author: acraig1@uci.edu

# Supplementary Tables

**Supplementary Table 1. Score – Blockwise Komolgorov Smirnov test statistic values.** The results of the two-sample Komolgorov-Smirnov (KS) hypothesis test comparing similarity between blocks as reported by KS test statistic.

| Block | 1 | 2 | 3 | 4 | 5 | 6 | 7 | 8 | 9 | 10 | 11 |
| --- | --- | --- | --- | --- | --- | --- | --- | --- | --- | --- | --- |
| 1 | 0.000 | 0.317 | 0.667 | 0.733 | 0.700 | 0.500 | 0.250 | 0.717 | 0.733 | 0.200 | 0.367 |
| 2 |  | 0.000 | 0.533 | 0.750 | 0.533 | 0.350 | 0.550 | 0.583 | 0.733 | 0.483 | 0.167 |
| 3 |  |  | 0.000 | 0.400 | 0.183 | 0.417 | 0.767 | 0.183 | 0.250 | 0.750 | 0.700 |
| 4 |  |  |  | 0.000 | 0.533 | 0.667 | 0.850 | 0.533 | 0.167 | 0.867 | 0.850 |
| 5 |  |  |  |  | 0.000 | 0.367 | 0.800 | 0.133 | 0.433 | 0.767 | 0.683 |
| 6 |  |  |  |  |  | 0.000 | 0.717 | 0.417 | 0.617 | 0.683 | 0.333 |
| 7 |  |  |  |  |  |  | 0.000 | 0.800 | 0.850 | 0.117 | 0.617 |
| 8 |  |  |  |  |  |  |  | 0.000 | 0.400 | 0.783 | 0.733 |
| 9 |  |  |  |  |  |  |  |  | 0.000 | 0.867 | 0.833 |
| 10 |  |  |  |  |  |  |  |  |  | 0.000 | 0.533 |
| 11 |  |  |  |  |  |  |  |  |  |  | 0.000 |

**Supplementary Table 2. Score – Blockwise Komolgorov Smirnov p-values.** The results of the two-sample Komolgorov-Smirnov (KS) hypothesis test comparing similarity between blocks as reported by p-value.

| Block | 1 | 2 | 3 | 4 | 5 | 6 | 7 | 8 | 9 | 10 | 11 |
| --- | --- | --- | --- | --- | --- | --- | --- | --- | --- | --- | --- |
| 1 | 1.000 | 0.004 | <0.001 | <0.001 | <0.001 | <0.001 | 0.039 | <0.001 | <0.001 | 0.160 | <0.001 |
| 2 |  | 1.000 | <0.001 | <0.001 | <0.001 | <0.001 | <0.001 | <0.001 | <0.001 | <0.001 | 0.345 |
| 3 |  |  | 1.000 | <0.001 | 0.239 | <0.001 | <0.001 | 0.239 | 0.039 | <0.001 | <0.001 |
| 4 |  |  |  | 1.000 | <0.001 | <0.001 | <0.001 | <0.001 | 0.345 | <0.001 | <0.001 |
| 5 |  |  |  |  | 1.000 | <0.001 | <0.001 | 0.629 | <0.001 | <0.001 | <0.001 |
| 6 |  |  |  |  |  | 1.000 | <0.001 | <0.001 | <0.001 | <0.001 | 0.002 |
| 7 |  |  |  |  |  |  | 1.000 | <0.001 | <0.001 | 0.784 | <0.001 |
| 8 |  |  |  |  |  |  |  | 1.000 | <0.001 | <0.001 | <0.001 |
| 9 |  |  |  |  |  |  |  |  | 1.000 | <0.001 | <0.001 |
| 10 |  |  |  |  |  |  |  |  |  | 1.000 | <0.001 |
| 11 |  |  |  |  |  |  |  |  |  |  | 1.000 |

**Supplementary Table 3. Score – Blockwise mean and standard deviation (in points).**

| Block | Mean | SD |
| --- | --- | --- |
| 1 | 34.667 | 41.213 |
| 2 | 28.433 | 28.727 |
| 3 | -2.867 | 19.472 |
| 4 | -19.900 | 16.099 |
| 5 | -1.300 | 21.201 |
| 6 | 14.967 | 22.439 |
| 7 | 52.200 | 30.480 |
| 8 | -0.500 | 16.367 |
| 9 | -16.600 | 17.335 |
| 10 | 51.600 | 27.651 |
| 11 | 31.233 | 21.691 |

**Supplementary Table 4. Score – Uncertainty level Komolgorov Smirnov test statistic values.** The results of the two-sample Komolgorov-Smirnov (KS) hypothesis test comparing similarity between uncertainty level as reported by KS test statistic.

| Uncertainty | No | Low | Mod | High |
| --- | --- | --- | --- | --- |
| No | 0.000 | 0.494 | 0.733 | 0.817 |
| Low |  | 0.000 | 0.522 | 0.733 |
| Mod |  |  | 0.000 | 0.400 |
| High |  |  |  | 0.000 |

**Supplementary Table 5. Score – Uncertainty level Komolgorov Smirnov p-values.** The results of the two-sample Komolgorov-Smirnov (KS) hypothesis test comparing similarity between uncertainty level as reported by p-value.

| Uncertainty | No | Low | Mod | High |
| --- | --- | --- | --- | --- |
| No | 1 | <0.001 | <0.001 | <0.001 |
| Low |  | 1 | <0.001 | <0.001 |
| Mod |  |  | 1 | <0.001 |
| High |  |  |  | 1 |

**Supplementary Table 6. Score – Uncertainty level mean and standard deviation (in points).**

| Uncertainty | Mean | SD |
| --- | --- | --- |
| No | 46.156 | 34.417 |
| Low | 24.878 | 25.372 |
| Mod | -1.556 | 19.037 |
| High | -18.250 | 16.740 |

**Supplementary Table 7. Observe use (all trials) – Uncertainty level Komolgorov Smirnov test statistic values.** The results of the two-sample Komolgorov-Smirnov (KS) hypothesis test comparing similarity between uncertainty levels as reported by KS test statistic.

| Uncertainty | No | Low | Mod | High |
| --- | --- | --- | --- | --- |
| No | 0.000 | 0.086 | 0.197 | 0.300 |
| Low |  | 0.000 | 0.117 | 0.214 |
| Mod |  |  | 0.000 | 0.125 |
| High |  |  |  | 0.000 |

**Supplementary Table 8. Observe use (all trials) – Uncertainty level Komolgorov Smirnov p-values.** The results of the two-sample Komolgorov-Smirnov (KS) hypothesis test comparing similarity between uncertainty levels as reported by p-value.

| Uncertainty | No | Low | Mod | High |
| --- | --- | --- | --- | --- |
| No | 1.000 | 0.640 | 0.006 | <0.001 |
| Low |  | 1.000 | 0.161 | 0.002 |
| Mod |  |  | 1.000 | 0.196 |
| High |  |  |  | 1.000 |

**Supplementary Table 9. Observe use (all trials) – Uncertainty level mean and standard deviation (in # observes).**

| Uncertainty | Mean [#] | SD |
| --- | --- | --- |
| No | 9.400 | 13.710 |
| Low | 9.706 | 12.855 |
| Mod | 11.789 | 13.219 |
| High | 15.042 | 14.908 |

**Supplementary Table 10. Observe use (by half block).** The results of the two-sample Komolgorov-Smirnov (KS) hypothesis test comparing similarity between uncertainty levels, mean and standard deviation of each half block (in proportion of trials using observe). The first block did not feature the option to observe.

| Block | Mean [1st] | Mean [2nd] | SD [1st] | SD [2nd] | P-value | KS-statistic |
| --- | --- | --- | --- | --- | --- | --- |
| 1 | N/A | N/A | N/A | N/A | N/A | N/A |
| 2 | 0.092 | 0.114 | 0.162 | 0.199 | 0.911 | 0.100 |
| 3 | 0.154 | 0.189 | 0.216 | 0.243 | 0.784 | 0.117 |
| 4 | 0.243 | 0.287 | 0.284 | 0.298 | 0.629 | 0.133 |
| 5 | 0.254 | 0.272 | 0.293 | 0.298 | 0.981 | 0.083 |
| 6 | 0.258 | 0.219 | 0.290 | 0.279 | 0.911 | 0.100 |
| 7 | 0.157 | 0.181 | 0.261 | 0.279 | 0.981 | 0.083 |
| 8 | 0.251 | 0.295 | 0.288 | 0.302 | 0.911 | 0.100 |
| 9 | 0.329 | 0.345 | 0.334 | 0.334 | 0.911 | 0.100 |
| 10 | 0.211 | 0.203 | 0.293 | 0.297 | 0.999 | 0.067 |
| 11 | 0.244 | 0.238 | 0.306 | 0.307 | 0.999 | 0.067 |

**Supplementary Table 11. Run of Observes – Blockwise Komolgorov Smirnov test statistic values.** The results of the two-sample Komolgorov-Smirnov (KS) hypothesis test comparing similarity between blocks as reported by KS test statistic. The first block did not feature the option to observe.

| Block | 1 | 2 | 3 | 4 | 5 | 6 | 7 | 8 | 9 | 10 | 11 |
| --- | --- | --- | --- | --- | --- | --- | --- | --- | --- | --- | --- |
| 1 | N/A | N/A | N/A | N/A | N/A | N/A | N/A | N/A | N/A | N/A | N/A |
| 2 |  | 0.000 | 0.063 | 0.308 | 0.348 | 0.407 | 0.351 | 0.392 | 0.332 | 0.295 | 0.360 |
| 3 |  |  | 0.000 | 0.301 | 0.346 | 0.348 | 0.291 | 0.366 | 0.307 | 0.297 | 0.335 |
| 4 |  |  |  | 0.000 | 0.127 | 0.140 | 0.084 | 0.164 | 0.104 | 0.055 | 0.135 |
| 5 |  |  |  |  | 0.000 | 0.119 | 0.164 | 0.101 | 0.133 | 0.128 | 0.154 |
| 6 |  |  |  |  |  | 0.000 | 0.138 | 0.109 | 0.130 | 0.140 | 0.188 |
| 7 |  |  |  |  |  |  | 0.000 | 0.173 | 0.131 | 0.121 | 0.159 |
| 8 |  |  |  |  |  |  |  | 0.000 | 0.102 | 0.113 | 0.146 |
| 9 |  |  |  |  |  |  |  |  | 0.000 | 0.107 | 0.126 |
| 10 |  |  |  |  |  |  |  |  |  | 0.000 | 0.154 |
| 11 |  |  |  |  |  |  |  |  |  |  | 0.000 |

**Supplementary Table 12. Run of Observes – Blockwise Komolgorov Smirnov p-values.** The results of the two-sample Komolgorov-Smirnov (KS) hypothesis test comparing similarity between blocks as reported by p-value. The first block did not feature the option to observe.

| Block | 1 | 2 | 3 | 4 | 5 | 6 | 7 | 8 | 9 | 10 | 11 |
| --- | --- | --- | --- | --- | --- | --- | --- | --- | --- | --- | --- |
| 1 | N/A | N/A | N/A | N/A | N/A | N/A | N/A | N/A | N/A | N/A | N/A |
| 2 |  | 1.000 | 1.000 | 0.029 | 0.015 | 0.002 | 0.022 | 0.003 | 0.018 | 0.058 | 0.011 |
| 3 |  |  | 1.000 | 0.024 | 0.011 | 0.010 | 0.071 | 0.005 | 0.026 | 0.043 | 0.015 |
| 4 |  |  |  | 1.000 | 0.853 | 0.751 | 0.999 | 0.549 | 0.953 | 1.000 | 0.794 |
| 5 |  |  |  |  | 1.000 | 0.929 | 0.690 | 0.982 | 0.832 | 0.884 | 0.708 |
| 6 |  |  |  |  |  | 1.000 | 0.864 | 0.961 | 0.849 | 0.805 | 0.443 |
| 7 |  |  |  |  |  |  | 1.000 | 0.612 | 0.888 | 0.946 | 0.731 |
| 8 |  |  |  |  |  |  |  | 1.000 | 0.973 | 0.950 | 0.756 |
| 9 |  |  |  |  |  |  |  |  | 1.000 | 0.964 | 0.873 |
| 10 |  |  |  |  |  |  |  |  |  | 1.000 | 0.708 |
| 11 |  |  |  |  |  |  |  |  |  |  | 1.000 |

**Supplementary Table 13. Run of Observes – Blockwise mean and standard deviation (in average # of observes per run).** The first block did not feature the option to observe.

| Block | Mean [length] | SD |
| --- | --- | --- |
| 1 | N/A | N/A |
| 2 | 3.469 | 8.093 |
| 3 | 2.571 | 3.011 |
| 4 | 3.842 | 4.613 |
| 5 | 3.708 | 4.158 |
| 6 | 4.224 | 7.022 |
| 7 | 4.423 | 5.818 |
| 8 | 5.189 | 10.478 |
| 9 | 5.678 | 10.225 |
| 10 | 5.506 | 10.797 |
| 11 | 6.544 | 13.136 |

**Supplementary Table 14. Run of Observes – Uncertainty level Komolgorov Smirnov test statistic values.** The results of the two-sample Komolgorov-Smirnov (KS) hypothesis test comparing similarity between uncertainty level as reported by KS test statistic. The first block did not feature the option to observe.

| Uncertainty | No | Low | Mod | High |
| --- | --- | --- | --- | --- |
| No | 0.000 | 0.181 | 0.138 | 0.093 |
| Low |  | 0.000 | 0.202 | 0.187 |
| Mod |  |  | 0.000 | 0.127 |
| High |  |  |  | 0.000 |

**Supplementary Table 15. Run of Observes – Uncertainty level Komolgorov Smirnov p-values.** The results of the two-sample Komolgorov-Smirnov (KS) hypothesis test comparing similarity between uncertainty level as reported by p-value. The first block did not feature the option to observe.

| Uncertainty | No | Low | Mod | High |
| --- | --- | --- | --- | --- |
| No | 1.000 | 0.780 | 0.927 | 0.998 |
| Low |  | 1.000 | 0.652 | 0.681 |
| Mod |  |  | 1.000 | 0.934 |
| High |  |  |  | 1.000 |

**Supplementary Table 16. Run of Observes – Uncertainty level mean and standard deviation (in average # of observes per run).** The first block did not feature the option to observe.

| Uncertainty | Mean | SD |
| --- | --- | --- |
| No | 4.673 | 6.959 |
| Low | 3.702 | 3.732 |
| Mod | 3.537 | 3.651 |
| High | 4.710 | 5.789 |

**Supplementary Table 17. Win Stay – Blockwise Komolgorov Smirnov test statistic values.** The results of the two-sample Komolgorov-Smirnov (KS) hypothesis test comparing similarity between blocks as reported by KS test statistic.

| Block | 1 | 2 | 3 | 4 | 5 | 6 | 7 | 8 | 9 | 10 | 11 |
| --- | --- | --- | --- | --- | --- | --- | --- | --- | --- | --- | --- |
| 1 | 0.000 | 0.359 | 0.224 | 0.427 | 0.333 | 0.233 | 0.300 | 0.317 | 0.283 | 0.291 | 0.371 |
| 2 |  | 0.000 | 0.220 | 0.475 | 0.428 | 0.197 | 0.081 | 0.127 | 0.262 | 0.109 | 0.097 |
| 3 |  |  | 0.000 | 0.339 | 0.229 | 0.097 | 0.232 | 0.147 | 0.107 | 0.279 | 0.256 |
| 4 |  |  |  | 0.000 | 0.127 | 0.295 | 0.506 | 0.403 | 0.265 | 0.520 | 0.538 |
| 5 |  |  |  |  | 0.000 | 0.267 | 0.444 | 0.353 | 0.233 | 0.487 | 0.464 |
| 6 |  |  |  |  |  | 0.000 | 0.239 | 0.162 | 0.083 | 0.268 | 0.260 |
| 7 |  |  |  |  |  |  | 0.000 | 0.155 | 0.276 | 0.068 | 0.071 |
| 8 |  |  |  |  |  |  |  | 0.000 | 0.190 | 0.185 | 0.162 |
| 9 |  |  |  |  |  |  |  |  | 0.000 | 0.308 | 0.287 |
| 10 |  |  |  |  |  |  |  |  |  | 0.000 | 0.098 |
| 11 |  |  |  |  |  |  |  |  |  |  | 0.000 |

**Supplementary Table 18. Win Stay – Blockwise Komolgorov Smirnov p-values.** The results of the two-sample Komolgorov-Smirnov (KS) hypothesis test comparing similarity between blocks as reported by p-value.

| Block | 1 | 2 | 3 | 4 | 5 | 6 | 7 | 8 | 9 | 10 | 11 |
| --- | --- | --- | --- | --- | --- | --- | --- | --- | --- | --- | --- |
| 1 | 1.000 | <0.001 | 0.087 | <0.001 | 0.002 | 0.064 | 0.007 | 0.004 | 0.014 | 0.011 | <0.001 |
| 2 |  | 1.000 | 0.098 | <0.001 | <0.001 | 0.177 | 0.988 | 0.708 | 0.029 | 0.865 | 0.938 |
| 3 |  |  | 1.000 | 0.002 | 0.075 | 0.928 | 0.073 | 0.519 | 0.873 | 0.017 | 0.038 |
| 4 |  |  |  | 1.000 | 0.693 | 0.009 | <0.001 | <0.001 | 0.026 | <0.001 | <0.001 |
| 5 |  |  |  |  | 1.000 | 0.022 | <0.001 | <0.001 | 0.069 | <0.001 | <0.001 |
| 6 |  |  |  |  |  | 1.000 | 0.058 | 0.389 | 0.984 | 0.024 | 0.033 |
| 7 |  |  |  |  |  |  | 1.000 | 0.454 | 0.019 | 0.999 | 0.998 |
| 8 |  |  |  |  |  |  |  | 1.000 | 0.222 | 0.252 | 0.411 |
| 9 |  |  |  |  |  |  |  |  | 1.000 | 0.006 | 0.014 |
| 10 |  |  |  |  |  |  |  |  |  | 1.000 | 0.939 |
| 11 |  |  |  |  |  |  |  |  |  |  | 1.000 |

**Supplementary Table 19. Win Stay – Blockwise mean and standard deviation (in proportion of stay trials after a winning trial).**

| Block | Mean | SD |
| --- | --- | --- |
| 1 | 0.846 | 0.254 |
| 2 | 0.890 | 0.211 |
| 3 | 0.862 | 0.216 |
| 4 | 0.732 | 0.257 |
| 5 | 0.745 | 0.281 |
| 6 | 0.832 | 0.243 |
| 7 | 0.896 | 0.218 |
| 8 | 0.903 | 0.184 |
| 9 | 0.833 | 0.238 |
| 10 | 0.920 | 0.171 |
| 11 | 0.918 | 0.201 |

**Supplementary Table 20. Win Stay – Uncertainty level Komolgorov Smirnov test statistic values.** The results of the two-sample Komolgorov-Smirnov (KS) hypothesis test comparing similarity between uncertainty level as reported by KS test statistic.

| Uncertainty | No | Low | Mod | High |
| --- | --- | --- | --- | --- |
| No | 0.000 | 0.131 | 0.228 | 0.361 |
| Low |  | 0.000 | 0.210 | 0.310 |
| Mod |  |  | 0.000 | 0.162 |
| High |  |  |  | 0.000 |

**Supplementary Table 21. Win Stay – Uncertainty level Komolgorov Smirnov p-values.** The results of the two-sample Komolgorov-Smirnov (KS) hypothesis test comparing similarity between uncertainty level as reported by p-value.

| Uncertainty | No | Low | Mod | High |
| --- | --- | --- | --- | --- |
| No | 1.000 | 0.089 | <0.001 | <0.001 |
| Low |  | 1.000 | <0.001 | <0.001 |
| Mod |  |  | 1.000 | 0.045 |
| High |  |  |  | 1.000 |

**Supplementary Table 22. Win Stay – Uncertainty level mean and standard deviation (in proportion of stay trials after a winning trial).**

| Uncertainty | Mean | SD |
| --- | --- | --- |
| No | 0.887 | 0.219 |
| Low | 0.879 | 0.221 |
| Mod | 0.835 | 0.239 |
| High | 0.782 | 0.252 |

**Supplementary Table 23. Lose Shift – Blockwise Komolgorov Smirnov test statistic values.** The results of the two-sample Komolgorov-Smirnov (KS) hypothesis test comparing similarity between blocks as reported by KS test statistic.

| Block | 1 | 2 | 3 | 4 | 5 | 6 | 7 | 8 | 9 | 10 | 11 |
| --- | --- | --- | --- | --- | --- | --- | --- | --- | --- | --- | --- |
| 1 | 0.000 | 0.200 | 0.150 | 0.217 | 0.217 | 0.180 | 0.144 | 0.198 | 0.215 | 0.119 | 0.131 |
| 2 |  | 0.000 | 0.267 | 0.367 | 0.350 | 0.363 | 0.167 | 0.331 | 0.348 | 0.169 | 0.222 |
| 3 |  |  | 0.000 | 0.200 | 0.167 | 0.163 | 0.294 | 0.125 | 0.188 | 0.206 | 0.100 |
| 4 |  |  |  | 0.000 | 0.117 | 0.119 | 0.310 | 0.092 | 0.173 | 0.302 | 0.228 |
| 5 |  |  |  |  | 0.000 | 0.070 | 0.263 | 0.086 | 0.156 | 0.254 | 0.224 |
| 6 |  |  |  |  |  | 0.000 | 0.259 | 0.108 | 0.136 | 0.298 | 0.229 |
| 7 |  |  |  |  |  |  | 0.000 | 0.293 | 0.277 | 0.088 | 0.199 |
| 8 |  |  |  |  |  |  |  | 0.000 | 0.132 | 0.260 | 0.204 |
| 9 |  |  |  |  |  |  |  |  | 0.000 | 0.281 | 0.226 |
| 10 |  |  |  |  |  |  |  |  |  | 0.000 | 0.130 |
| 11 |  |  |  |  |  |  |  |  |  |  | 0.000 |

**Supplementary Table 24. Lose Shift – Blockwise Komolgorov Smirnov p-values.** The results of the two-sample Komolgorov-Smirnov (KS) hypothesis test comparing similarity between blocks as reported by p-value.

| Block | 1 | 2 | 3 | 4 | 5 | 6 | 7 | 8 | 9 | 10 | 11 |
| --- | --- | --- | --- | --- | --- | --- | --- | --- | --- | --- | --- |
| 1 | 1.000 | 0.160 | 0.477 | 0.103 | 0.103 | 0.263 | 0.543 | 0.177 | 0.112 | 0.794 | 0.679 |
| 2 |  | 1.000 | 0.022 | <0.001 | <0.001 | <0.001 | 0.351 | 0.002 | 0.001 | 0.363 | 0.104 |
| 3 |  |  | 1.000 | 0.160 | 0.345 | 0.374 | 0.009 | 0.720 | 0.218 | 0.159 | 0.926 |
| 4 |  |  |  | 1.000 | 0.784 | 0.770 | 0.005 | 0.956 | 0.309 | 0.009 | 0.089 |
| 5 |  |  |  |  | 1.000 | 0.998 | 0.028 | 0.975 | 0.432 | 0.042 | 0.099 |
| 6 |  |  |  |  |  | 1.000 | 0.032 | 0.862 | 0.618 | 0.010 | 0.089 |
| 7 |  |  |  |  |  |  | 1.000 | 0.011 | 0.018 | 0.976 | 0.193 |
| 8 |  |  |  |  |  |  |  | 1.000 | 0.659 | 0.037 | 0.171 |
| 9 |  |  |  |  |  |  |  |  | 1.000 | 0.018 | 0.097 |
| 10 |  |  |  |  |  |  |  |  |  | 1.000 | 0.725 |
| 11 |  |  |  |  |  |  |  |  |  |  | 1.000 |

**Supplementary Table 25. Lose Shift – Blockwise mean and standard deviation (in proportion of shift trials after a losing trial).**

| Block | Mean | SD |
| --- | --- | --- |
| 1 | 0.748 | 0.174 |
| 2 | 0.801 | 0.168 |
| 3 | 0.719 | 0.166 |
| 4 | 0.694 | 0.126 |
| 5 | 0.680 | 0.177 |
| 6 | 0.695 | 0.157 |
| 7 | 0.763 | 0.206 |
| 8 | 0.695 | 0.159 |
| 9 | 0.659 | 0.193 |
| 10 | 0.745 | 0.208 |
| 11 | 0.722 | 0.198 |

**Supplementary Table 26. Lose Shift – Uncertainty level Komolgorov Smirnov test statistic values.** The results of the two-sample Komolgorov-Smirnov (KS) hypothesis test comparing similarity between uncertainty level as reported by KS test statistic.

| Uncertainty | No | Low | Mod | High |
| --- | --- | --- | --- | --- |
| No | 0.000 | 0.117 | 0.204 | 0.243 |
| Low |  | 0.000 | 0.143 | 0.199 |
| Mod |  |  | 0.000 | 0.090 |
| High |  |  |  | 0.000 |

**Supplementary Table 27. Lose Shift – Uncertainty level Komolgorov Smirnov p-values.** The results of the two-sample Komolgorov-Smirnov (KS) hypothesis test comparing similarity between uncertainty level as reported by p-value.

| Block | No | Low | Mod | High |
| --- | --- | --- | --- | --- |
| No | 1.000 | 0.177 | 0.001 | <0.001 |
| Low |  | 1.000 | 0.051 | 0.006 |
| Mod |  |  | 1.000 | 0.585 |
| High |  |  |  | 1.000 |

**Supplementary Table 28. Lose Shift – Uncertainty level mean and standard deviation (in proportion of shift trials after a losing trial).**

| Uncertainty | Mean | SD |
| --- | --- | --- |
| No | 0.752 | 0.195 |
| Low | 0.740 | 0.180 |
| Mod | 0.698 | 0.168 |
| High | 0.676 | 0.163 |

**Supplementary Table 29. Probability Matching (all trials) – Blockwise t-test statistics.** The results of the one-sample t-test comparing similarity between proportion of feature selection and ground truth as reported by t statistic, degrees of freedom, mean, standard deviation, and p-value.

| Rule | Block | T-stat | DF | Mean | SD | P-value |
| --- | --- | --- | --- | --- | --- | --- |
| Top | 1 | -12.279 | 59 | 0.673 | 0.206 | <0.001 |
| Top | 2 | -8.765 | 59 | 0.710 | 0.168 | <0.001 |
| Top | 3 | -10.339 | 59 | 0.574 | 0.132 | <0.001 |
| Top | 4 | -8.044 | 59 | 0.452 | 0.143 | <0.001 |
| Top | 5 | -7.588 | 59 | 0.568 | 0.186 | <0.001 |
| Top | 6 | -9.060 | 59 | 0.684 | 0.185 | <0.001 |
| Top | 7 | -7.800 | 59 | 0.794 | 0.204 | <0.001 |
| Top | 8 | -7.183 | 57 | 0.606 | 0.153 | <0.001 |
| Top | 9 | -4.860 | 58 | 0.491 | 0.172 | <0.001 |
| Top | 10 | -8.065 | 57 | 0.818 | 0.172 | <0.001 |
| Top | 11 | -5.340 | 56 | 0.773 | 0.180 | <0.001 |
| Mid | 1 | 11.025 | 59 | 0.159 | 0.112 | <0.001 |
| Mid | 2 | 6.236 | 59 | 0.140 | 0.086 | <0.001 |
| Mid | 3 | 3.398 | 59 | 0.235 | 0.080 | 0.001 |
| Mid | 4 | 1.222 | 59 | 0.316 | 0.102 | 0.227 |
| Mid | 5 | 3.224 | 59 | 0.251 | 0.123 | 0.002 |
| Mid | 6 | 6.888 | 59 | 0.175 | 0.118 | <0.001 |
| Mid | 7 | 6.954 | 59 | 0.105 | 0.117 | <0.001 |
| Mid | 8 | 2.898 | 57 | 0.239 | 0.102 | 0.005 |
| Mid | 9 | 0.006 | 58 | 0.300 | 0.155 | 0.995 |
| Mid | 10 | 9.239 | 57 | 0.078 | 0.064 | <0.001 |
| Mid | 11 | 3.734 | 56 | 0.111 | 0.082 | <0.001 |
| Bottom | 1 | 10.220 | 59 | 0.168 | 0.127 | <0.001 |
| Bottom | 2 | 9.709 | 59 | 0.150 | 0.096 | <0.001 |
| Bottom | 3 | 12.229 | 59 | 0.191 | 0.090 | <0.001 |
| Bottom | 4 | 9.350 | 59 | 0.232 | 0.110 | <0.001 |
| Bottom | 5 | 8.508 | 59 | 0.181 | 0.119 | <0.001 |
| Bottom | 6 | 8.504 | 59 | 0.142 | 0.102 | <0.001 |
| Bottom | 7 | 5.488 | 59 | 0.100 | 0.142 | <0.001 |
| Bottom | 8 | 9.112 | 57 | 0.155 | 0.088 | <0.001 |
| Bottom | 9 | 5.741 | 58 | 0.209 | 0.146 | <0.001 |
| Bottom | 10 | 6.173 | 57 | 0.105 | 0.129 | <0.001 |
| Bottom | 11 | 4.535 | 56 | 0.117 | 0.144 | <0.001 |

**Supplementary Table 30. Probability Matching (all trials) – Uncertainty level t-test statistics.** The results of the one-sample t-test comparing similarity between proportion of feature selection and ground truth as reported by t statistic, degrees of freedom, mean, standard deviation, and p-value.

| Rule | Uncertainty | T-stat | DF | Mean | SD | P-value |
| --- | --- | --- | --- | --- | --- | --- |
| Top | No | -15.618 | 177 | 0.761 | 0.204 | <0.001 |
| Top | Low | -13.168 | 176 | 0.721 | 0.180 | <0.001 |
| Top | Mod | -14.117 | 177 | 0.582 | 0.159 | <0.001 |
| Top | High | -8.856 | 118 | 0.471 | 0.159 | <0.001 |
| Mid | No | 14.422 | 177 | 0.114 | 0.106 | <0.001 |
| Mid | Low | 9.609 | 76 | 0.142 | 0.100 | <0.001 |
| Mid | Mod | 5.413 | 177 | 0.242 | 0.103 | <0.001 |
| Mid | High | 0.684 | 118 | 0.308 | 0.130 | 0.496 |
| Bottom | No | 12.240 | 177 | 0.124 | 0.136 | <0.001 |
| Bottom | Low | 12.251 | 176 | 0.136 | 0.116 | <0.001 |
| Bottom | Mod | 16.708 | 177 | 0.176 | 0.101 | <0.001 |
| Bottom | High | 10.224 | 118 | 0.221 | 0.129 | <0.001 |

**Supplementary Table 31. Probability Matching (last 10 trials of block) – Blockwise t-test statistics.** The results of the one-sample t-test comparing similarity between proportion of feature selection and ground truth as reported by t statistic, degrees of freedom, mean, standard deviation, and p-value.

| Rule | Block | T-stat | DF | Mean | SD | P-value |
| --- | --- | --- | --- | --- | --- | --- |
| Top | 1 | -13.559 | 48 | 0.940 | 0.031 | <0.001 |
| Top | 2 | -0.255 | 51 | 0.897 | 0.075 | 0.800 |
| Top | 3 | 3.350 | 44 | 0.813 | 0.126 | 0.002 |
| Top | 4 | -2.759 | 53 | 0.529 | 0.189 | 0.008 |
| Top | 5 | 2.430 | 46 | 0.805 | 0.154 | 0.019 |
| Top | 6 | 0.098 | 49 | 0.901 | 0.083 | 0.922 |
| Top | 7 | -6.690 | 50 | 0.975 | 0.027 | <0.001 |
| Top | 8 | 3.250 | 44 | 0.820 | 0.144 | 0.002 |
| Top | 9 | 0.721 | 53 | 0.623 | 0.238 | 0.474 |
| Top | 10 | -6.579 | 52 | 0.973 | 0.030 | <0.001 |
| Top | 11 | 2.152 | 51 | 0.920 | 0.068 | 0.036 |
| Mid | 1 | 8.013 | 48 | 0.028 | 0.025 | <0.001 |
| Mid | 2 | -2.908 | 51 | 0.050 | 0.049 | 0.005 |
| Mid | 3 | -5.148 | 44 | 0.108 | 0.120 | <0.001 |
| Mid | 4 | -0.181 | 53 | 0.295 | 0.186 | 0.857 |
| Mid | 5 | -5.263 | 46 | 0.117 | 0.109 | <0.001 |
| Mid | 6 | -2.724 | 49 | 0.049 | 0.054 | 0.009 |
| Mid | 7 | 5.871 | 50 | 0.018 | 0.022 | <0.001 |
| Mid | 8 | -5.813 | 44 | 0.098 | 0.118 | <0.001 |
| Mid | 9 | -1.305 | 53 | 0.259 | 0.233 | 0.198 |
| Mid | 10 | 4.314 | 52 | 0.011 | 0.018 | <0.001 |
| Mid | 11 | -6.168 | 51 | 0.035 | 0.041 | <0.001 |
| Bottom | 1 | 7.340 | 48 | 0.032 | 0.031 | <0.001 |
| Bottom | 2 | 3.739 | 51 | 0.052 | 0.043 | <0.001 |
| Bottom | 3 | 3.181 | 44 | 0.080 | 0.062 | 0.003 |
| Bottom | 4 | 4.415 | 53 | 0.176 | 0.126 | <0.001 |
| Bottom | 5 | 2.166 | 46 | 0.079 | 0.091 | 0.036 |
| Bottom | 6 | 2.115 | 49 | 0.049 | 0.065 | 0.040 |
| Bottom | 7 | 3.105 | 50 | 0.007 | 0.016 | 0.003 |
| Bottom | 8 | 2.067 | 44 | 0.082 | 0.105 | 0.045 |
| Bottom | 9 | 1.248 | 53 | 0.118 | 0.106 | 0.217 |
| Bottom | 10 | 4.602 | 52 | 0.017 | 0.027 | <0.001 |
| Bottom | 11 | 2.147 | 51 | 0.045 | 0.051 | 0.037 |

**Supplementary Table 32. Probability Matching (last 10 trials of block) – Uncertainty level t-test statistics.** The results of the one-sample t-test comparing similarity between proportion of feature selection and ground truth as reported by t statistic, degrees of freedom, mean, standard deviation, and p-value.

| Rule | Uncertainty | T-stat | DF | Mean | SD | P-value |
| --- | --- | --- | --- | --- | --- | --- |
| Top | No | -13.813 | 152 | 0.963 | 0.033 | <0.001 |
| Top | Low | 1.030 | 153 | 0.906 | 0.076 | 0.305 |
| Top | Mod | 5.167 | 136 | 0.812 | 0.141 | <0.001 |
| Top | High | -1.129 | 107 | 0.576 | 0.219 | 0.261 |
| Mid | No | 10.237 | 152 | 0.019 | 0.023 | <0.001 |
| Mid | Low | -6.487 | 153 | 0.045 | 0.048 | <0.001 |
| Mid | Mod | -9.412 | 136 | 0.108 | 0.115 | <0.001 |
| Mid | High | -1.133 | 107 | 0.277 | 0.211 | 0.260 |
| Bottom | No | 8.456 | 152 | 0.018 | 0.027 | <0.001 |
| Bottom | Low | 4.418 | 153 | 0.049 | 0.053 | <0.001 |
| Bottom | Mod | 4.050 | 136 | 0.080 | 0.087 | <0.001 |
| Bottom | High | 4.079 | 107 | 0.147 | 0.119 | <0.001 |
